# Circulating Fatty Acid Synthase in pregnant women: Relationship to blood pressure, maternal metabolism and newborn parameters

**DOI:** 10.1038/srep24167

**Published:** 2016-04-19

**Authors:** Gemma Carreras-Badosa, Anna Prats-Puig, Teresa Puig, Montserrat Vázquez-Ruíz, Monserrat Bruel, Ericka Mendoza, Francis de Zegher, Lourdes Ibáñez, Abel López-Bermejo, Judit Bassols

**Affiliations:** 1 Pediatrics, Girona Institute for Biomedical Research, 17007 Girona, Spain; 2 Pediatrics, Dr. Josep Trueta Hospital, 17007 Girona, Spain; 3 TargetsLab, Medical Sciences Department, Faculty of Medicine, University of Girona, 17003 Girona, Spain; 4 Pediatrics, Salut Empordà Foundation, 17600 Figueres, Spain; 5 Obstetrics and Gynecology, Salut Empordà Foundation, 17600 Figueres, Spain; 6 Department of Development & Regeneration, University of Leuven, 3000 Leuven, Belgium; 7 Pediatric Endocrinology Unit, Sant Joan de Déu Children’s Hospital, 08950 Esplugues, Barcelona; 8 CIBERDEM (Center for Network Biomedical Research in Diabetes and Related Metabolic Diseases), ISCIII, Madrid, Spain

## Abstract

The enzyme FASN (fatty acid synthase) is potentially related with hypertension and metabolic dysfunction. *FASN* is highly expressed in the human placenta. We aimed to investigate the relationship circulating FASN has with blood pressure, maternal metabolism and newborn parameters in healthy pregnant women. Circulating FASN was assessed in 115 asymptomatic pregnant women in the second trimester of gestation along with C-peptide, fasting glucose and insulin, post-load glucose lipids, HMW-adiponectin and blood pressure (the latter was assessed in each trimester of gestation). At birth, newborns and placentas were weighed. *FASN* expression was also able to be assessed in 80 placentas. Higher circulating FASN was associated with lower systolic blood pressure (SBP), with a more favourable metabolic phenotype (lower fasting glucose and insulin, post load glucose, HbAc1, HOMA-IR and C-peptide), and with lower placental and birth weight (all p < 0.05 to p < 0.001). Placental *FASN* expression related positively to circulating FASN (p < 0.005) and negatively to placental weight (p < 0.05). Our observations suggest a physiological role of placental FASN in human pregnancy. Future studies will clarify whether circulating FASN of placental origin does actually regulate placental and fetal growth, and (thereby) has a favourable influence on the pregnant mother’s insulin sensitivity and blood pressure.

The multifunctional protein complex FASN (fatty acid synthase) is indispensable in the synthesis of saturated straight-chain fatty acids from acetyl coenzyme A (CoA), via malonyl-CoA^1^. Excess energy intake and increased insulin levels has the effect of upregulating the *FASN* gene expression^2^,^3^; suggesting this enzyme is implicated in energy homeostasis.

Altered *FASN* activity/expression has been reported in metabolic syndrome and overweight subjects who exhibit obesity, inflammation, hypertension, insulin resistance, dyslipidemia and atherosclerosis, indicating a relationship between FASN and the pathogenesis of hypertension and metabolic dysfunction^4^,^5^. Adipose tissue from hypertensive individuals showed decreased levels of *FASN* mRNA^6^. The subcutaneous adipose tissue of the obese subjects also showed decreased *FASN* expression compared to lean subjects^7^,^8^,^9^,^10^,^11^, and has exhibited negative correlation with insulin resistance markers such as glucose, HbA1c and HOMA-IR^6^,^7^,^8^. In adipose tissue of insulin resistant type 2 diabetic patients, *FASN* mRNA expression is markedly decreased in response to reduced insulin signalling^12^.

*In vivo*, the cellular concentrations of poly-unsaturated fatty acids and sterols are the main factors regulating the expression of *FASN*, when their concentrations decrease in the cell, the transcription of the *FASN* gene increases. FASN can be actively removed out of the cell when AMPK (adenosine monophosphate-activated protein kinase) is activated^9^. Circulating FASN is thus thought to reflect previous intracellular enzymatic activity^9^.

In normal cells, low levels of FASN are present due to abundant dietary lipids. However, *FASN* is highly expressed in hepatic, adipose tissue and in neoplastic cells, where *FASN* expression and the synthesis of new fatty acids are up-regulated as a survival advantage to low-fuel supply^13^,^14^. The placenta also expresses high amounts of *FASN*^15^,^16^. Trophoblastic cells may use *de novo* lipid synthesis in order to maintain essential placental actions for development. This strategy may also be an evolutionary favoured compensatory mechanism, as the lipid supply from food intake may become limited during pregnancy.

The role of FASN in human pregnancy is poorly studied. A recent report indicates that maternal obesity and gestational diabetes are related to less expression of *FASN* in adipose tissue of subcutaneous and visceral origin^17^. In mice with a lipid-poor diet during gestation, an augmented expression of *FASN* in adipose tissue was reported^18^.

Despite the core physiological role of FASN in maintaining normal levels of lipids and glucose, as well as energy homeostasis and its high expression in placenta, the relationship between the circulating form of this molecule, blood pressure and metabolism during human pregnancy has not been characterized. In this work, we studied the associations of circulating FASN with blood pressure, maternal metabolism and newborn parameters in normal human pregnancy. We also studied whether circulating FASN was related to placental *FASN* expression.

## Results

Table 1 summarizes the clinical and laboratory findings of the study subjects.

### Correlation analyses

Second- and third-trimester SBP decreased with increasing circulating FASN. Higher circulating FASN was also related to a more favourable metabolic condition, specifically lower fasting glucose and insulin, post load glucose, HbAc1, HOMA-IR and C-peptide (all p < 0.05 to p < 0.001; Table 2). Circulating FASN was inversely related to placental weight and birth weight (p < 0.05 to p < 0.01; Table 2), and directly associated with placental *FASN* mRNA expression (p < 0.001; Table 2). Placental *FASN* expression was also inversely related to the weight of the placenta (p < 0.05), however, it was unrelated to blood pressure or to metabolic parameters in pregnant women (data not shown).

### Tertiles of circulating FASN

**Figure 1:**
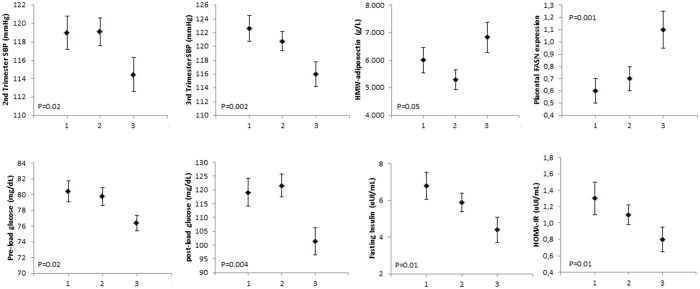
Distribution of metabolic parameters: blood pressure [second- and third-trimester systolic blood pressure (SBP)], HMW-adiponectin, pre- and post-load glucose, insulin, HOMA-IR and placental *FASN* expression according to tertiles of circulating FASN (1st: 0.1–1.4 ng/ml; 2nd: 1.4–4.3 ng/ml and 3^rd^: 4.5–18.8 ng/ml). Data are means and SEM.

A threshold association was evident for circulating FASN and BP, metabolic variables and the parameters of newborns, with women in the highest tertile of circulating FASN exhibiting lower SBP in the second and third trimester of gestation, DBP in the third trimester, fasting glucose and insulin, post load glucose HOMA-IR, placental FASN expression, placental weight and birth weight SDS, and higher HMW-adiponectin compared to other tertiles of circulating FASN (Table 3 and Fig. 1 and Suppl Fig. 1).

### Multiple regression analyses

In multiple regression analyses (Table 4), circulating FASN remained independently related to the second- (β = −0.231, p = 0.008) and third- (β = −0.333, p < 0.001) trimester SBP, fasting and post-load glucose (β = −0.204, p = 0.028 and β = −0.261, p = 0.004, respectively), HOMA-IR (β = −0.257, p = 0.006), and placental and birth weight (β = −0.214, p = 0.030 and β = −0.194, p = 0.015, respectively), even when confounding variables where introduced in the model.

Placental *FASN* expression also remained correlated to placental weight (β = −0.238, p = 0.047) independently of confounding variables in multivariate analyses (Table 5).

## Discussion

Higher circulating FASN was associated with a more favourable blood pressure and metabolic profile, with lower placental weight and birth weight and with higher placental *FASN* expression in healthy pregnant women.

The independent associations of circulating FASN with blood pressure and with glucose- and insulin-related markers concur with previous reports in overweight and hypertensive subjects that showed decreased *FASN* expression in their fat depots^6^,^19^. Additionally, a negative relation between *FASN* expression and insulin resistance markers, has been reported in adipose tissue of healthy subjects without diabetes^6^,^8^. Evidence, however, exists for an increased *FASN* expression and higher circulating FASN in obesity-related disorders^5^,^20^. These discrepancies may be explained, at least in part, by the apparently opposing effects of insulin on *FASN*, causing both long-term upregulation of *FASN* expression and short-term *FASN* inactivation; the latter being dependent on the insulinemic state of the individual^21^. Our study was based on healthy pregnant women with no comorbidities. These associations may vary in patients with chronic insulin resistance including patients with vascular or metabolic comorbidities and long-standing obesity.

Although the metabolic regulation of FASN is not yet completely understood, these current results suggest that circulating FASN may be among the factors integrating blood pressure regulation and energy metabolism during gestation. AMPK may provide a plausible molecular mechanism for these associations. Into the cell, FASN activity is limited by the activation of the nuclear AMPK enzyme that removes FASN from the cytosolic milieu into the extracellular space^9^. AMPK activation is also known to protect mice from developing hypertension^22^ and metabolic disease.

Preeclampsia is a systemic disorder of pregnancy originating in the placenta. Research has demonstrated that several placental antiangiogenic factors are liberated into circulation during pregnancy and cause widespread endothelial dysfunction, hypertension and other systemic manifestations of preeclampsia^23^. Besides adipose tissue, *FASN* is highly expressed in normal trophoblastic cells^15^. *FASN* provides trophoblasts with an alternative mechanism to maintain a regular supply of fatty acids to keep up with the metabolic demands of this organ. Changes in FASN activity may thus cause trophoblastic cell dysfunction which may, in turn, affect the regulation of blood pressure and energy metabolism during gestation. Recent data indicate that *FASN* expression in human placenta is influenced by maternal cholesterolemia and glycemia^16^. We do not exclude the possibility that mRNA *FASN* levels may be also associated with metabolic parameters in pregnant women with comorbidities, such as gestational diabetes, hypertension or dyslipidemia. Our present results suggest a better correlation of protein rather than mRNA levels with energy metabolism in healthy pregnant women.

Our results showed negative associations of placental weight and/or birth weight with circulating FASN and placental *FASN* expression. The human placenta is comprised by a trophoblast layer that physically limits maternal and fetal blood flows. Maternal plasma lipoproteins cross the placenta to supply the fetus with lipids^24^. Fetal tissues and the placenta also synthetize fatty acids and cholesterol to compensate for a possible lipid deficiency during pregnancy^25^.

Therefore, we would suggest that the changes in *FASN* expression in smaller placentas may be a compensatory mechanism to regulate maternal blood pressure and metabolic parameters in normal pregnancies with lower fetoplacental growth.

Increased *FASN* expression may regulate the fatty acid outflow from other tissues, such as adipose tissue, preventing placentas from being too big or too small, as well as contributing to an improvement in the physiological insulin-resistant state that is observed during pregnancy. In this sense, during pregnancy, to compensate for a poor lipid diet, several adaptations of the maternal metabolism may be the increasing expression of those genes implicated in the synthesis of fatty acids (among them *FASN)* in the maternal tissues including liver, adipose tissue and the mammary glands^18^.

We are aware of some limitations in our study. A cause-effect relationship cannot be described between circulating FASN and blood pressure or metabolic variables in pregnant women by the present study design. The exact link between circulating FASN and blood pressure in women with pathological conditions, such as preeclampsia or metabolic disorders cannot be discerned in our healthy study population. Moreover, circulating FASN was taken at a single point in the second trimester. Serial measurements of FASN throughout the pregnancy and in maternal hypertensive or dysglycemic conditions would have added greater value to our present results.

In conclusion, circulating FASN was associated with a more favourable blood pressure and metabolic profile and with lower placental and birth weight. Circulating FASN and placental *FASN* expression were closely related. Future studies will clarify whether circulating FASN of placental origin regulates placental and fetal growth, and (thereby) has a favourable effect on blood pressure and insulin sensitivity in the pregnant mother.

## Methods

### Study population and ethics

115 pregnant Caucasian women experiencing normal pregnancies and delivering normal birth weight infants were included in the study. These infants were included in a prenatal cohort of healthy infants. The women were recruited at the Figueres Hospital^26^ and none of the women experienced complicated pregnancies or parturition. Women with complications, such as preeclampsia, gestational diabetes, fetal malformations, asphyxia and multiple pregnancies were excluded from this prenatal cohort of apparently healthy mothers and babies.

The Dr. Josep Trueta Hospital’s Institutional Review Board approved the protocol. All the women signed an informed written consent. The methods were implemented according to approved guidelines.

### Assessments

All patients were subjected to clinical examinations, ultrasonograms and blood tests. Mothers gave information about socio-demographic characteristics, medical data during pregnancy and at delivery. Anthropometric measurements were derived from standard medical records^27^.

The weight, height, BMI (in Kg/m^2^) and blood pressure (both systolic and diastolic) of the pregnant women were assessed at each trimester of gestation. An electronic sphygmomanometer (Dinamap Pro 100, GE Healthcare, UK) was used on the right arm and with women in the sitting position to determine the blood pressure.

All infants were born between 37 and 42 weeks and had normal birth weights (from −2.0 to +2.0 DE). Within the first hour after delivery, infants were measured with a measuring board and weighed with a calibrated scale. Birth weight and length were adjusted for gestational age and sex according to regional norms^28^.

### Analytical methods

All serum samples for assessing circulating FASN and metabolic markers were obtained under fasting conditions between 24 and 28 gestation weeks, when glucose tolerance assessment was carried out. All women were subjected to fasting glucose and oral glucose tolerance test (1 h with 50 g glucose).

The hexokinase method was used to analyse serum glucose. To determine HbA1c the ion-exchange HPLC method was used (Bio-Rad Laboratories, S.A. Madrid, Spain). Immuno-chemiluminiscence was used to measure serum insulin (IMMULITE 2000, Siemens Healthcare, Madrid, Spain) with a detection limit of 0.4 mIU/L and coefficients of variability less than 10%. HOMA-IR was calculated according to the formula: fasting insulin (mU/l) × fasting glucose (mM)/22.5. High-molecular-weight adiponectin was determined with enzyme-linked immunosorbent assay (Linco Research, Missouri, USA) with a limit of detection of 0.5 ng/mL and coefficients of variability less than 4%.

Serum lipids including triglycerides and HDL cholesterol were determined using routine laboratory tests. A quantitatie ELISA was used to measure serum FASN concentrations (FASgen Diagnostics, Baltimore, USA) with a limit of detection of 0.3 ng/ml and coefficients of variability less than 12%.

### Placental collection

Placental tissue was studied in a representative subgroup (n = 80) of the women studied and who did not differ in clinical or laboratory parameters from the whole group. Placentas were weighed at the moment of delivery. Three fragments of ~1 cm^3^ of tissue were biopsied on the maternal side for each placenta. The samples were embedded in RNAlater (QIAGEN, Madrid, Spain) into cryotubes and stored at −80 °C until RNA extraction.

### Gene expression

Placental biopsies were each homogenized and total RNA was extracted (RNeasy Mini Kit; QIAGEN, Madrid, Spain) using the standard protocol with DNase. The absorbance at 260 nm was used for assessing RNA concentration (Spectramax Plus 384 Microplate Reader, Molecular Devices, Berkshire, UK). A260/A280 ratio was used to exclude protein contamination.

One microgram of RNA was converted to cDNA following the instructions of the manufacturer (High-Capacity RNA-to-cDNA Kit, Life Technologies SA, Madrid, Spain). To perform real time PCR 25 μl mixtures were prepared containing 2xTaqman Universal Master Mix (12.5 μl) (Applied Biosystems), cDNA (4 ng/μl), and the following Taqman Gene Expression Assays (1.25 μl) (Life Technologies SA, Madrid, Spain): FASN (Hs00188012), TBP (Hs99999910) and SDHA (Hs00360422). The cycling protocol was 50 °C, 2 min; 95 °C, 10 min; then 40 cycles of 95 °C, 15 s and 60 °C, 1 min (ABI PRISM® 7000 Sequence Detection System; Life Technologies SA, Madrid, Spain). The 2−ΔΔCT method was used to calculate the relative expression using the Ct values of the housekeeping genes TBP and SDHA^27^.

### Statistics

Results are expressed as mean ± SEM. Statistical analyses were performed using SPSS statistics software (IBM, Madrid, Spain). Logarithmic transformation was used to restore symmetry of non-parametric variables. Pearson correlation test and multiple regression analyses in a stepwise manner were used to analyze the association between quantitative variables. Variables of interest were also analysed by One-Way ANOVA according to tertiles of circulating FASN and p < 0.05 was used as the significance level. The study exhibited a power of 80% and a 0.26 Pearson’s correlation coefficient to detect a significant association between serum FASN, blood pressure and metabolic parameters, and a 0.30 Pearson’s correlation coefficient between serum FASN and placental FASN expression.

## Additional Information

**How to cite this article**: Carreras-Badosa, G. *et al*. Circulating Fatty Acid Synthase in pregnant women: Relationship to blood pressure, maternal metabolism and newborn parameters. *Sci. Rep*. **6**, 24167; doi: 10.1038/srep24167 (2016).

## Supplementary Material

Supplementary Information

## Figures and Tables

**Table 1:** Clinical and laboratory assessments in healthy pregnant women.

Clinical assessments	1^st^ Trimester	2^nd^ Trimester	3^rd^ Trimester
n	115	115	115
Age (yr)	30.2 ± 0.2	–	–
Height (m)	163 ± 1	–	–
Weight (Kg)	66 ± 1	72 ± 1	77 ± 1
BMI (Kg/m^2^)	25 ± 1	27 ± 1	29 ± 1
SBP (mm Hg)	116 ± 1	117 ± 1	119 ± 1
DBP (mm Hg)	68 ± 1	69 ± 1	72 ± 1
Laboratory variables			
Pre-load glucose (mg/dL)	–	78 ± 1	–
Post-load glucose (mg/dL)	–	113 ± 3	–
HbA1C (%)	–	5.0 ± 0.1	–
Fasting insulin (μIU/mL)	–	6.0 ± 0.5	–
HOMA-IR	–	1.2 ± 0.1	–
C-peptide	–	1.6 ± 0.1	–
HMW-adiponectin (mg/L)	–	6.1 ± 0.2	–
Triacylglycerol (mg/dL)	–	161 ± 1	–
Total cholesterol (mg/dL)	–	257 ± 2	–
HDL cholesterol (mg/dL)	–	71 ± 1	–
LDL cholesterol (mg/dL)	–	154 ± 2	–
Circulating FASN (ng/mL)	–	4.4 ± 0.2	–
Placental and Newborns’ Parameters			
Placental *FASN* expression^a^	–	–	0.8 ± 0.1
Placental weight (g)	–	–	603 ± 8
Birth weight (g)	–	–	3300 ± 30
Birth length (cm)	–	–	49.5 ± 0.1
Birth weight SDS	–	–	0.1 ± 0.1
Birth length SDS	–	–	−0.1 ± 0.1

Data are shown as mean ± SEM. BMI: body mass index; SBP & DBP: systolic and diastolic blood pressure; HOMA-IR: homeostasis model assessment insulin resistance; FASN: fatty acid synthase.

^a^Assessed after parturition in 80 women.

**Table 2:** Correlation analyses of circulating FASN with clinical and laboratory parameters in healthy pregnant women.

Clinical assessments	1^st^ Trimester	2^nd^ Trimester	3^rd^ Trimester
Age (yr)	0.020	–	–
Height (m)	−0.162	–	–
Weight (Kg)	−0.149	−0.142	−0.137
BMI (Kg/m^2^)	−0.085	−0.069	−0.062
SBP (mm Hg)	−0.107	−0.256**	−0.280***
DBP (mm Hg)	−0.175	−0.116	−0.210*
Laboratory variables			
Pre-load glucose (mg/dL)	–	−0.227*	–
Post-load glucose (mg/dL)	–	−0.298***	–
HbA1C (%)	–	−0.225*	–
Fasting insulin (μIU/ml)	–	−0.274**	–
HOMA-IR	–	−0.285**	–
C-peptide (ng/mL)	–	−0.210*	–
HMW-adiponectin (mg/L)	–	0.144	–
Triacylglycerol (mg/dL)	–	0.011	–
Total cholesterol (mg/dL)	–	0.016	–
HDL cholesterol (mg/dL)	–	−0.037	–
LDL cholesterol (mg/dL)	–	0.020	–
Placental and Newborns’ Parameters			
Placental *FASN* expression^a^	–	–	0.348***
Placental weight (g)	–	–	−0.201*
Birth weight (g)	–	–	−0.194*
Birth length (cm)	–	–	−0.151
Birth weight SDS	–	–	−0.247**
Birth length SDS	–	–	−0.174

BMI: body mass index; SBP & DBP: systolic and diastolic blood pressure; HOMA-IR: homeostasis model assessment insulin resistance; FASN: fatty acid synthase.

^a^Assessed after parturition in 80 women. *p < 0.05, **p < 0.01 and ***p < 0.005 from Pearson correlations.

**Table 3:** Clinical and laboratory assessments in healthy pregnant women according to tertiles of circulating FASN.

Clinical assessments	Circulating FASN tertiles		
	0.1–1.4 ng/ml	1.4–4.3 ng/ml	4.5–18.8 ng/ml
n	38	39	38
Age (yr)	30 ± 1	29 ± 1	31 ± 1
Height (m)	163 ± 1	163 ± 1	162 ± 1
1^st^ trimester			
BMI (Kg/m^2^)	26 ± 1	25 ± 1	24 ± 1
SBP (mm Hg)	118 ± 2	119 ± 2	114 ± 2
DBP (mm Hg)	71 ± 1	70 ± 1	67 ± 2
2^nd^ trimester			
BMI (Kg/m^2^)	28 ± 1	27 ± 1	26 ± 1
SBP (mm Hg)	119 ± 2	120 ± 1	114 ± 2*
DBP (mm Hg)	70 ± 1	69 ± 2	68 ± 1
3^rd^ trimester			
BMI (Kg/m^2^)	30 ± 1	29 ± 1	28 ± 1
SBP (mm Hg)	123 ± 2	121 ± 2	116 ± 2**
DBP (mm Hg)	75 ± 1	72 ± 1	70 ± 2*
Laboratory variables			
Pre-load glucose (mg/dL)	80 ± 1	80 ± 1	76 ± 1*
Post-load glucose (mg/dL)	119 ± 5	122 ± 4	101 ± 5**
HbA1C (%)	5.1 ± 0.1	5.0 ± 0.1	4.9 ± 0.1
Fasting insulin (μIU/mL)	6.8 ± 0.8	5.9 ± 0.5	4.4 ± 0.7**
HOMA-IR	1.3 ± 0.2	1.1 ± 0.1	0.8 ± 0.1**
C-peptide	1.6 ± 0.1	1.6 ± 0.1	1.4 ± 0.1
HMW-adiponectin (g/L)	5.8 ± 0.4	4.9 ± 0.3	6.8 ± 0.5*
Triacylglycerol (mg/dL)	160 ± 11	175 ± 9	153 ± 8
Total cholesterol (mg/dL)	251 ± 8	268 ± 8	254 ± 9
HDL cholesterol (mg/dL)	68 ± 2	74 ± 2	69 ± 3
LDL cholesterol (mg/dL)	151 ± 7	160 ± 7	153 ± 7
Circulating FASN (ng/mL)	0.7 ± 0.1	2.8 ± 0.1	10.1 ± 1***
Placental and Newborns’ Parameters			
Placental *FASN* expression^a^	0.6 ± 0.1	0.7 ± 0.1	1.1 ± 0.1***
Placental weight (g)	625 ± 25	593 ± 20	585 ± 23*
Birth weight (g)	3382 ± 53	3272 ± 50	3256 ± 47
Birth length (cm)	50 ± 0.3	50 ± 0.2	49 ± 0.2
Birth weight SDS	0.2 ± 0.1	0.1 ± 0.1	−0.1 ± 0.1*
Birth length SDS	−0.1 ± 0.2	−0.1 ± 0.2	−0.3 ± 0.1

Data are shown as mean ± SEM. BMI: body mass index; SBP & DBP: systolic and diastolic blood pressure; HOMA-IR: homeostasis model assessment insulin resistance; FASN: fatty acid synthase.

^a^Assessed after parturition in 80 women. *p < 0.05, **p < 0.01 and ***p < 0.001 from One-Way ANOVA.

**Table 4:** Multivariate linear models of circulating FASN and gestational SBP, metabolic variables and newborn’s parameters in healthy pregnant women.

**(a)**									
**SBP (mmHg)**	**2**^**nd**^ **Trimester**			**3**^**rd**^ **Trimester**					
	**Beta**	**Sig.**	**R**^**2**^	**Beta**	**Sig.**	**R**^**2**^			
BMI (Kg/m^2^)	0.373	<0.001	15.1%	0.258	0.005	4.1%			
FASN (ng/ml)	−0.231	0.008	5.3%	−0.333	<0.001	8.1%			
**(b)**									
**Metabolic Variables**	**Pre-load Glucose**			**Post-load Glucose**			**HOMA-IR**		
	**Beta**	**Sig.**	**R**^**2**^	**Beta**	**Sig.**	**R**^**2**^	**Beta**	**Sig.**	**R**^**2**^
BMI (Kg/m^2^)	0.257	0.006	7.7%	0.200	0.027	3.4%	0.262	0.005	8.1%
FASN (ng/ml)	−0.204	0.028	4.7%	−0.261	0.004	7.2%	−0.257	0.006	6.6%
HOMA-IR (uIU/ml)	0.187	0.044	3.5%	–	–	–	–	–	–
**(c)**									
**Newborns’ Parameters**	**Placental Weight**			**Birth Weight**					
	**Beta**	**Sig.**	**R**^**2**^	**Beta**	**Sig.**	**R**^**2**^			
BMI (Kg/m^2^)	0.247	0.020	9.2%	–	–	–			
FASN (ng/ml)	−0.214	0.030	3.9%	−0.194	0.015	3.8%			
Gestational age	0.219	0.027	4.2%	0.610	<0.001	37.4%			

(**a**) Non-predictive variables: age, HbA1c, HOMA-IR and serum lipids. (**b**) Non-predictive variables: age and serum lipids. (**c**) Non-predictive variables: sex, maternal age, pre- or post-load glucose, HbA1c, HOMA-IR and serum lipids.

**Table 5:** Multivariate linear models of placental *FASN* expression and placental weight in healthy pregnant women.

	Placental Weight		
	Beta	Sig.	R^2^
Placental *FASN* expression^a^	−0.238	0.047	5.6%
Gestational age	0.323	0.008	10.6%

Non-predictive variables: sex, maternal age and BMI, pre- or post-load glucose, HbA1c, HOMA-IR and serum lipids.

^a^Assessed after parturition in 80 women.
