# Supplementary material for: Circulating Fatty Acid Synthase in pregnant women: Relationship to blood pressure, maternal metabolism and newborn parameters
Source: Sci Rep. 2016 Apr 19;6:24167. doi: 10.1038/srep24167 (PMC4835700; doi:10.1038/srep24167)
Supplement: Supplementary Information [file srep24167-s1.doc]

**Circulating Fatty Acid Synthase in pregnant women: Relationship to blood pressure, maternal metabolism and newborn parameters.**

Gemma Carreras-Badosa BS ^1,2^ , Anna Prats-Puig **PhD** ^1,2^ , Teresa Puig **PhD** ^3^,

Montserrat Vázquez-Ruíz RN ^4^, Monserrat Bruel RN ^5^, Ericka Mendoza RN ^5^,

Francis de Zegher **MD, PhD** ^6^, Lourdes Ibáñez **MD, PhD** ^7,8^ ,

Abel Lopez-Bermejo **MD*** ^1,2^ , Judit Bassols **PhD***^1,2^

**Supplementary Figure 1.** Distribution of newborn parameters: placental weight and birth weight SDS according to tertiles of circulating FASN (1st: 0.1-1.4 ng/ml; 2nd: 1.4-4.3 ng/ml and 3^rd^: 4.5-18.8 ng/ml). Data are means and SEM.
